# Influenza A (H3) illness and viral aerosol shedding from symptomatic naturally infected and experimentally infected cases

**DOI:** 10.1111/irv.12790

**Published:** 2020-07-23

**Authors:** Paul Jacob Bueno de Mesquita, Jonathan Nguyen‐Van‐Tam, Ben Killingley, Joanne Enstone, Robert Lambkin‐Williams, Anthony S. Gilbert, Alexander Mann, John Forni, Jing Yan, Jovan Pantelic, Michael L. Grantham, Donald K. Milton

**Affiliations:** ^1^ University of Maryland School of Public Health Maryland Institute for Applied Environmental Health College Park MD USA; ^2^ Division of Epidemiology and Public Heath Health Protection and Influenza Research Group University of Nottingham School of Medicine Nottingham UK; ^3^ hVIVO London UK; ^4^ Present address: virologyconsult.com; ^5^ Present address: Department of Acute and Specialty Care MSD London UK; ^6^ Present address: Center for the Built Environment University of California Berkeley CA USA; ^7^ Present address: Missouri Western State University St. Joseph MO USA

**Keywords:** experimental inoculation, human challenge model, influenza symptomatology, influenza transmission, propensity scores, Viral aerosols, viral shedding

## Abstract

**Background:**

It has long been known that nasal inoculation with influenza A virus produces asymptomatic to febrile infections. Uncertainty persists about whether these infections are sufficiently similar to natural infections for studying human‐to‐human transmission.

**Methods:**

We compared influenza A viral aerosol shedding from volunteers nasally inoculated with A/Wisconsin/2005 (H3N2) and college community adults naturally infected with influenza A/H3N2 (2012‐2013), selected for influenza‐like illness with objectively measured fever or a positive Quidel QuickVue A&B test. Propensity scores were used to control for differences in symptom presentation observed between experimentally and naturally infected groups.

**Results:**

Eleven (28%) experimental and 71 (86%) natural cases shed into fine particle aerosols (*P* < .001). The geometric mean (geometric standard deviation) for viral positive fine aerosol samples from experimental and natural cases was 5.1E + 3 (4.72) and 3.9E + 4 (15.12) RNA copies/half hour, respectively. The 95th percentile shedding rate was 2.4 log_10_ greater for naturally infected cases (1.4E + 07 vs 7.4E + 04). Certain influenza‐like illness‐related symptoms were associated with viral aerosol shedding. The almost complete lack of symptom severity distributional overlap between groups did not support propensity score–adjusted shedding comparisons.

**Conclusions:**

Due to selection bias, the natural and experimental infections had limited symptom severity distributional overlap precluding valid, propensity score–adjusted comparison. Relative to the symptomatic naturally infected cases, where high aerosol shedders were found, experimental cases did not produce high aerosol shedders. Studying the frequency of aerosol shedding at the highest observed levels in natural infections without selection on symptoms or fever would support helpful comparisons.

## 1 INTRODUCTION

There is uncertainty about the extent to which the site of initial exposure within the pulmonary tree influences influenza virus infection risk and severity between humans. Experimental nasal instillation produces a range of illness from asymptomatic, symptomatic, to febrile. Studies that challenged humans by nasal instillation of virus, and others that challenged with aerosolized virus suggest that upper respiratory mucosal exposure, as opposed to airborne exposure may result in a higher proportion of milder, afebrile illnesses.^1^, ^2^, ^3^ Anisotropic infection is defined by Milton as infection whereby transmission mode influences illness presentation,^4^ and has been used to characterize human influenza.^5^ To minimize health risk associated with experimental human influenza infection, the majority of human challenge models have adopted viral inoculation by nasal instillation.^6^ Associations between symptomatology and nasal and throat mucosal viral load following symptom onset have been reported among volunteers receiving intranasal influenza virus challenge and among secondary household cases in Hong Kong.^7^, ^8^, ^9^ Other analyses of these household transmission data did not find temporal associations between symptom severity and upper respiratory viral load^10^; and observed upper respiratory mucosal viral loads^11^, ^12^ or respiratory symptoms^13^ to be poorly predictive of transmission to household secondary cases, suggesting that other biomarkers of contagion such as exhaled breath aerosols should be explored. The current study compares fine aerosol shedding between influenza A/H3 nasally inoculated and naturally infected cases to test whether experimental, nasal‐induced infections have similar risk and rate of fine aerosol shedding compared with natural cases infected by any mode.

## 2 METHODS

### 2.1 Study design overview

Study design and data collection procedures for the Evaluating Modes of Influenza Transmission (EMIT) human challenge‐transmission trial^14^ and the observational study of naturally infected influenza cases from University of Maryland campus community are described elsewhere (SI Appendix S1).^15^ Half‐hour exhaled breath specimens are partitioned into fine (≤5 µm) and coarse (>5 µm) aerosol fractions during collection by Gesundheit‐II bioaerosol sampler (G‐II).^16^ Exhaled breath from both studies was evaluated using standard CDC qRT‐PCR primers and probes at the University of Maryland laboratory. Exhaled breath samples were taken on up to three consecutive days among naturally infected cases, and on up to 3 times over 4 days for experimental cases. Nasopharyngeal swabs from experimental and natural cases were evaluated with the same reagents in separate laboratories. Nasopharyngeal swabs from the experimental group were not tested against a plasmid standard for experimental cases, limiting comparison of RNA shedding in swabs to the cycle threshold (Ct) values. Data were cleaned and analyzed in R (v3.5.1 R Development Core Team, Vienna, Austria) and SAS Studio (Release 3.7 [Enterprise Edition], v9.4M6).

### 2.2 Symptom scores

Symptom scores were measured three times per day for experimental cases and once per day for natural cases during a research clinic visit where exhaled breath was collected. Scores taken closest in time to exhaled breath collection were selected for analysis. The upper respiratory score was sum of runny nose, stuffy nose, sneezing, sore throat, and earache symptom scores (range 0‐15). The lower respiratory score was the sum of shortness of breath and cough scores (range 0‐6). The systemic symptom score was the sum of malaise, headache, and muscle/join ache scores (range 0‐9). The tympanic temperature for experimental and oral for naturally infected cases was recorded. Observed cough counts were recorded during half‐hour breath collections.

### 2.3 Adjustment for qRT‐PCR detection limit

Tobit regression was used to impute fine aerosol RNA copy number for qRT‐PCR replicates below detection limit where one or more replicates for a sample had detectable RNA. Imputation of RNA copies was not done for samples without any replicates above detection limit, differing from the approach used by Yan and colleagues, where there were a minority of fine aerosol samples below detection limit (14%).^15^ It is less reasonable to do the same for the experimentally infected population where 72% of the observations would be imputed. Tobit regression imputed values for samples with qRT‐PCR detectable RNA in ≥1 replicate. For both experimentally and naturally infected populations, Tobit models consisted of fixed effects of cough and study day with random effect of person. Fixed effects for these models were selected based on a priori evidence of an association with fine aerosol shedding.^15^

### 2.4 Statistics and models to predict shedding

*t* Tests with equal variances and chi‐squared tests were used to compare continuous and categorical demographic and symptom severity variables. Fisher's exact tests were used to compare binomial proportions between experimental and natural cases for fine and coarse shedding subjects and samples. Welsh's *t* test for unequal variance and the Wilcoxon rank sum test were used to compare geometric mean (GM) and median aerosol shedding, respectively. Tests were two‐tailed. Unadjusted effects on probability of shedding into aerosols were estimated with a random effect of person (from generalized linear mixed‐effects model) for symptom scores, observed cough count, age, sex, and vaccination status. Analysis of aerosol shedding risk used all exhaled breath observations. Analysis of shedding quantity used the same predictors refined to the maximum shedding day per shedder.

### 2.5 Case selection and propensity adjustment

Naturally infected cases were sampled from a symptomatic population, selected on the basis of positive QuickVue^®^ rapid test or febrile illness >37.8°C (measured at University Health Center where some recruitment took place, or upon presentation to research clinic) plus cough or sore throat, and included in analysis based on a single qRT‐PCR–positive nasopharyngeal swab on the day of enrollment. Although enrollment of naturally infected cases used a febrile illness threshold of >37.8°C, this analysis used the more widely accepted threshold of >37.9°C. Experimentally infected cases were selected on qRT‐PCR detection of virus from nasopharyngeal swabs on at least two of six follow‐up days, or on one day plus serological evidence of infection. Differences in study design were expected to introduce imbalance in symptom severity distribution between groups (SI Appendices S1,S2). If symptoms are associated with aerosol shedding in an unselected population, then this would be an important variable to control for with the goal of assessing differences in shedding between the groups. To minimize the effect of this bias, we attempted to balance covariate distributions between populations with propensity score models (SI Appendix S3).

### 2.6 Data availability statement

The study data for experimental volunteers are available in the public repository at Nottingham University at https://rdmc.nottingham.ac.uk/handle/internal/8311 (DOI: http://doi.org/10.17639/nott.7051). The study data for natural infections are available upon request. All analysis scripts and readme files required to reproduce analyses are available at https://gitlab.com/jacobbueno/natural_vs_artificial_infection

## 3 RESULTS

For 39 experimental and 83 naturally infected influenza A H3 cases, there were 84 and 146 exhaled breath collection instances, respectively. Of the 39 confirmed experimental cases, 36 were qRT‐PCR positive two or more days, 31 of whom also had serological evidence of infection; three were qRT‐PCR positive on one day only and had serological evidence of infection. A total of 52 challenge study volunteers were inoculated, giving an infection rate of 75%, based on the current case infection definition.

Both study populations of young adults were generally healthy. The experimental group was on average 10 years older than the naturally infected. Experimental cases were more likely to be male, while naturally infected cases were balanced by sex (Table 1). Experimental cases had illness mostly characterized by upper respiratory symptoms or were asymptomatic (12.8%) (Table 1). There were small peaks in upper respiratory, lower respiratory, systemic, and cough scores, and cough counts on day 3 post‐inoculation. Naturally infected cases had more severe symptoms scores and greater cough counts, with symptom scores peaking on day 1 post‐symptom onset and aligning with day 3 post‐inoculation (Figure S2).

**Table 1:** Demographics and symptomatology of influenza A Cases

	Experimental	Natural	*P* value
N‐Participants	39	83	
Age	29.9 (7.0)	22.3 (7.6)	6E‐7
Range	20.0‐45.0	15.0‐63.0	
N‐Female (%)	11 (28.2)	47 (56.6)	.003
N‐asymptomatic (%)^a^	5 (12.8)	0 (0.0)	.003
N‐with fever > 37.9°C (%)	6 (15.4)	36 (43.4)	.002
N‐with flu vaccine in current yr (%)	0 (0)	14 (16.8)	E‐4
N‐with flu vaccine in current and previous yr (%)	0 (0)	10 (12.0)	.001
N‐Breath collection visits	84	146	
Temperature in C	36.6 (0.6)	37.3 (0.6)	4E‐16
N‐Missing observations	2	1	
Range	35.3‐38.4	36.3‐39.7	
Upper respiratory symptom score	1.4 (1.9)	7.0 (3.0)	E‐22
Range	0.0‐6.0	0.0‐15.0	
Lower respiratory symptom score	0.2 (0.4)	3.2 (1.5)	E‐22
Range	0.0‐1.0	0.0‐6.0	
Systemic symptom score	0.7 (1.2)	5.4 (2.4)	E‐22
Range	0.0‐5.0	0.0‐9.0	
Total symptom score	2.2 (2.9)	15.5 (5.5)	E‐22
Range	0.0‐11.0	4.0‐29.0	
Cough symptom score^b^	0.2 (0.4)	2.2 (0.8)	E‐22
Range	0.0‐1.0	0.0‐3.0	
Cough count^c^	1.7 (4.8)	26.7 (32.5)	3E‐11
Range	0.0‐35.0	0.0‐265.0	
Nasopharyngeal swab Ct value^d^	29.9 (6.9)	23.1 (6.1)	E‐7
N‐Missing observations	2	0	
Range	17.0‐40.0	13.1‐40.0	

Note

Mean (SD) unless indicated (N = number). Symptom scores, body temperature, and observed cough counts are reported per breath collection visit, with multiple visits per person.

^a^ Never fever >37.9°C or any self‐reported symptom.

^b^ Cough symptom score is part of composite lower respiratory score.

^c^ Cough counts after imputation for five experimental and three natural case observations.

^d^ Swabs with no detection were coded as having Ct value = 40.

**Figure 1:**
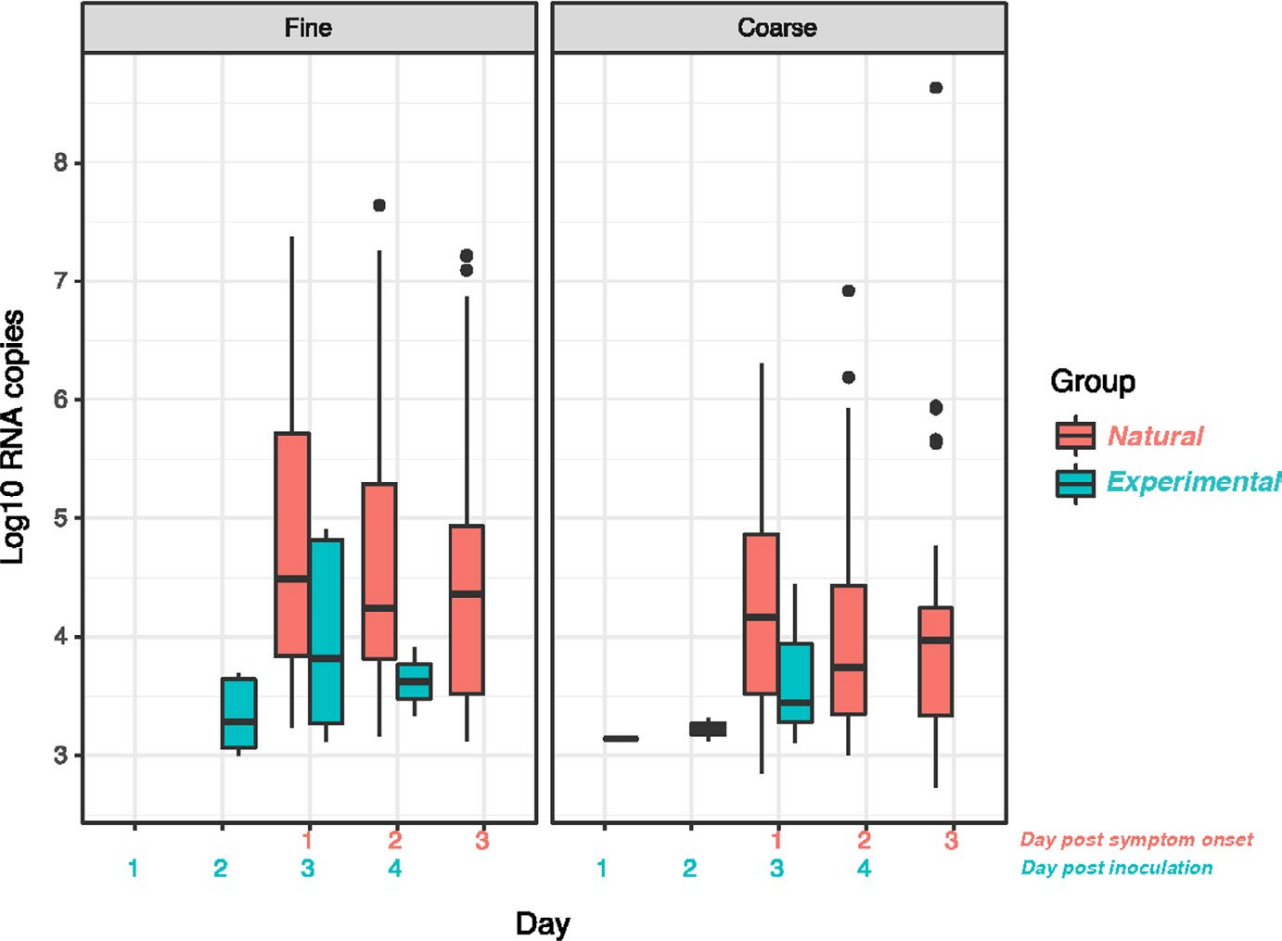
Aerosol shedding over time. Includes all observations (N = 84 experimental and N = 146 natural). Detectable aerosol shedding in log_10_ aerosol copies, with boxes showing the interquartile range (IQR) with a band to indicate the median, and whiskers extending to the highest and lowest data points within 1.5 IQR

The risk of shedding virus into coarse and fine aerosols for experimentally infected was 6/39 (15%) and 11/39 (28%), and for naturally infected 45/83 (54%) and 71/83 (86%) (Table 2). Median coarse and fine aerosol shedding quantity between the groups was significantly higher for natural cases in both fine (*P* < .001) and coarse (*P* < .001) aerosol fractions. Peak aerosol shedding was observed on day 3 post‐inoculation and day 1 post‐symptom onset for experimental and natural cases, respectively, matching peak symptom score day alignment (Figure 1). No virus was detected in fine aerosols on day 1 post‐inoculation in experimental cases. On the day of peak aerosol shedding, median symptom scores for experimental infection were upper respiratory 4 (IQR 2, 5), lower respiratory 0 (0, 1), systemic 1 (0, 2), cough 0 (0, 1), and cough count 0 (0, 6) and for natural infections 7 (5, 9), 3 (2, 4), 6 (4, 8), 2 (2, 3), and 22 (8, 40), respectively.

**Table 2:** Viral shedding into exhaled breath aerosols for experimental and natural infections

All exhaled breath observations						
	Experimental		Natural		*P* value	
	(39 subjects; 84 GII obs.)		(83 subjects; 146 GII obs.)			
	Coarse	Fine	Coarse	Fine	Coarse	Fine
No. of positive subjects (%)	6 (15)	11 (28)	45 (54)	71 (86)	6E‐5	7E‐10
No. of positive samples (%)	6 (7)	14 (17)	66 (45)	111 (76)	2E‐10	7E‐19
RNA copy GM (GSD)^a^	2.7E + 3 (3.3)	5.1E + 3 (4.7)	1.8E + 4 (13.9)	3.9E + 4 (15.1)	.002 (*t* = −3.9, *df* = 12.9)	.005 (*t* = −3.3, *df* = 16.1)
RNA copies by percentile						
25th	ND	ND	ND	1.3E + 3		
Median	ND	ND	ND	7.9E + 3	E‐9^b^	3E‐18^b^
75th	ND	ND	6.6E + 3	7.6E + 4		
90th	ND	2.0E + 3	7.4E + 4	1.1E + 6		
95th	1.3E + 3	6.4E + 3	8.5E + 5	6.5E + 6		
Maximum	2.8E + 4	8.0E + 4	4.3E + 8	4.4E + 7		

Maximum shedding exhaled breath observations						
	Experimental		Natural		*P* value	
	(11 subjects; 11 GII obs.)		(71 subjects; 71 GII obs.)			
	Coarse	Fine	Coarse	Fine	Coarse	Fine
RNA copy GM (GSD)	2.7E + 3 (3.3)	5.0E + 3 (5.8)	2.1E + 4 (16.5)	5.1E + 4 (17.0)	0.0002 (*t* = −4.2, *df* = 35.9)	0.003 (*t* = −3.5, *df* = 14.7)
RNA copies by percentile						
25th	ND	1.5E + 3	ND	5.8E + 3		
Median	ND	2.0E + 3	1.7E + 3	2.2E + 4	.02	.003
75th	ND	3.5E + 4	1.0E + 4	3.9E + 5		
90th	1.4E + 3	6.7E + 4	4.2E + 5	6.3E + 6		
95th	2.2E + 3	7.4E + 4	9.8E + 5	1.4E + 7		
Maximum	2.8E + 4	8.0E + 4	4.3E + 8	4.4E + 7		

Note

Samples collected days 1‐4 post‐inoculation in the experimentally infected and days 1‐3 post‐symptom onset in the naturally infected. Shedding per half‐hour sample.

Abbreviations: *df*, degrees of freedom; GM, geometric mean; GSD, geometric standard deviation; ND, not detected; *t*, *t* test value.

^a^ Only samples with detectable RNA (at least one positive replicate) contributed to GM and GSD (fine aerosol: N = 14 experimental, N = 111 natural; coarse aerosol: N = 6 experimental, N = 66 natural).

^b^ When restricted to samples with detectable RNA, Wilcoxon rank sum tests gave *P* = .03 (coarse) and *P* = .003 (fine).

When using all the detectable qRT‐PCR nasopharyngeal swab samples from days 1‐6 post‐inoculation (N = 179) and 1‐3 post‐symptom onset (N = 143), Ct values were notably lower for natural compared with experimental cases (Figure S3). The lowest Ct values were seen on day 1 post‐symptom onset in naturally infected cases and day 3 post‐inoculation; subsequent days showed a faster rise in Ct for experimental compared with natural cases.

**Figure 2:**
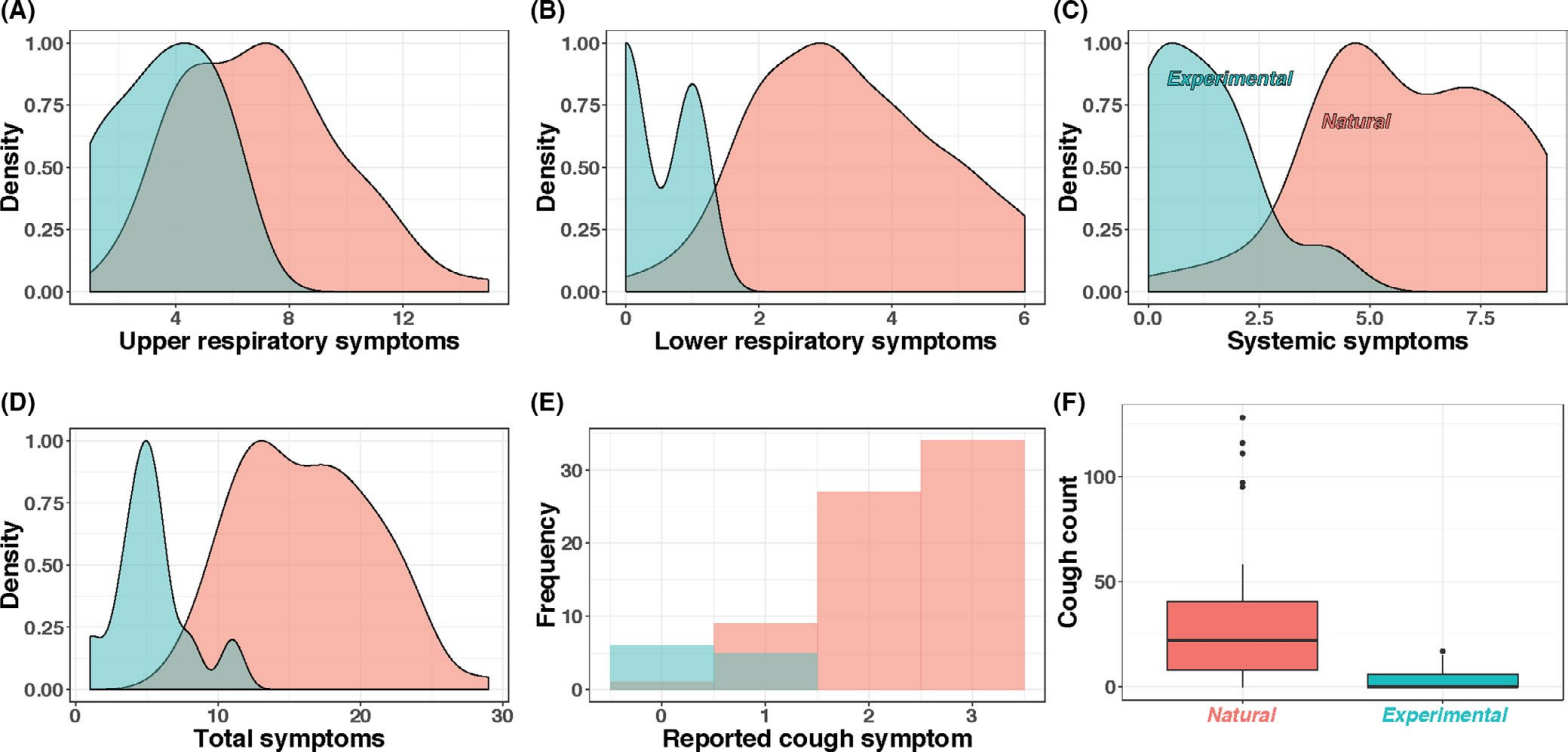
Comparison of self‐reported symptoms and observed coughs from maximum fine aerosol shedding days (N = 11 experimental (blue), N = 71 natural (red) observations). Cough counts with boxes showing the inner‐quartile range (IQR) with a band to indicate the median, and whiskers extending to the highest and lowest data points within 1.5 IQR

Restricted to maximum shedding observations by aerosol fraction, the GM (geometric standard deviation, GSD) for coarse and fine aerosols was 2.7E + 3 (3.3) and 5.1E + 3 (4.7) for experimentally, and 2.1E + 4 (16.5) and 5.1E + 4 (17.0) for naturally infected cases (Table 2, Figure S4). Descriptive statistics for covariates during maximum fine aerosol shedding observation days (Table S3) are similar to those derived from all observation days. Upper respiratory symptoms score distributions overlapped the most between groups, while differences in the distributions of lower respiratory, systemic, and cough symptoms scores, and cough count were more pronounced (Figure 2).

For the experimental cases, unadjusted upper and lower respiratory scores, cough symptoms, and cough count were positively associated with fine aerosol shedding detection (Table 3). For naturally infected cases, unadjusted lower respiratory symptom scores and cough symptoms were positively associated with fine aerosol shedding risk. Study day was negatively associated with aerosol shedding in naturally infected cases. Nasopharyngeal swab qRT‐PCR cycle threshold (Ct) value had a negative association with aerosol shedding risk for both groups. There were no significant predictors of aerosol shedding rate among experimental or natural cases. Of the experimentally infected, only males shed detectable virus into aerosols. Of the naturally infected, 57/69 (82.6%) unvaccinated and 14/14 (100%) vaccinated with the current influenza season vaccine shed into aerosols above detection limit, while 61/73 with and 10/10 without vaccination for the current and previous influenza seasonal vaccine shed virus into aerosols at measurable levels.

**Table 3:** Unadjusted odds ratios on shedding detectable virus into fine aerosols

Predictor	Experimental OR (95% CI)	Natural OR (95% CI)
Age	1.03 (0.94‐1.14)	0.95 (0.70‐1.27)
Sex^a^	–	2.61 (0.65‐10.43)
Ever fever > 37.9°C	3.12 (0.52‐18.67)	0.73 (0.21‐2.49)
Study day^b^		
Day 1	–	1.00 (REF)
Day 2	1.00 (REF)	0.25 (0.06‐1.03)
Day 3	50.89 (0.07‐30,636)	**0.17 (0.04‐0.73)**
Day 4	0 (0‐7.51)	–
Body temperature (^o^C)	4.77 (0.94‐24.17)	1.88 (0.87‐4.06)
Upper respiratory score	**2.24 (1.07‐4.69)**	1.07 (0.93‐1.22)
Lower respiratory score	**6.49 (1.14‐37.09)**	**1.35 (1.04‐1.76)**
Systemic symptom score	1.73 (0.94‐3.16)	1.15 (0.97‐1.36)
Total symptom score	**1.64 (1.09‐2.47)**	1.07 (0.99‐1.15)
Cough symptom score^c^		
No symptom	1.00 (REF)	1.00 (REF)
Mild	**7.49 (1.28‐43.91)**	8.5 (0.81‐88.85)
Moderate	–	**12.92 (1.32‐126.08)**
Severe	–	**20.4 (2.06‐202.21)**
Cough count	**1.29 (1.02‐1.62)**	1.92 (1‐3.65)
Nasopharyngeal swab Ct	**0.74 (0.6‐0.92)**	0.98 (0.91‐1.05)

Note

Symptom ORs (odds ratios) used data from N = 84 experimental and N = 146 natural observations (all exhaled breath observations and accompanying symptom data from 39 experimental and 83 natural cases). ORs for age, sex, ever fever > 37.9°C, used data from N = 39 experimental, and N = 83 natural cases. Effect of a single unit increase in age, body temperature, symptoms scores, and nasopharyngeal swab Ct; effect of interquartile range (IQR) increase in cough count, ever fever > 37.9°C vs afebrile, male vs female. Bold: significant at *P* < .05. Random effect of person.

^a^ Only males shed into aerosols in the experimental group.

^b^ Day post‐nasal inoculation for experimental (range 1‐4); day post‐symptom onset for natural (range 1‐3). No fine aerosol shedding was observed on day 1 post‐inoculation.

^c^ Moderate or severe cough was never observed in the experimental group.

**Figure 3:**
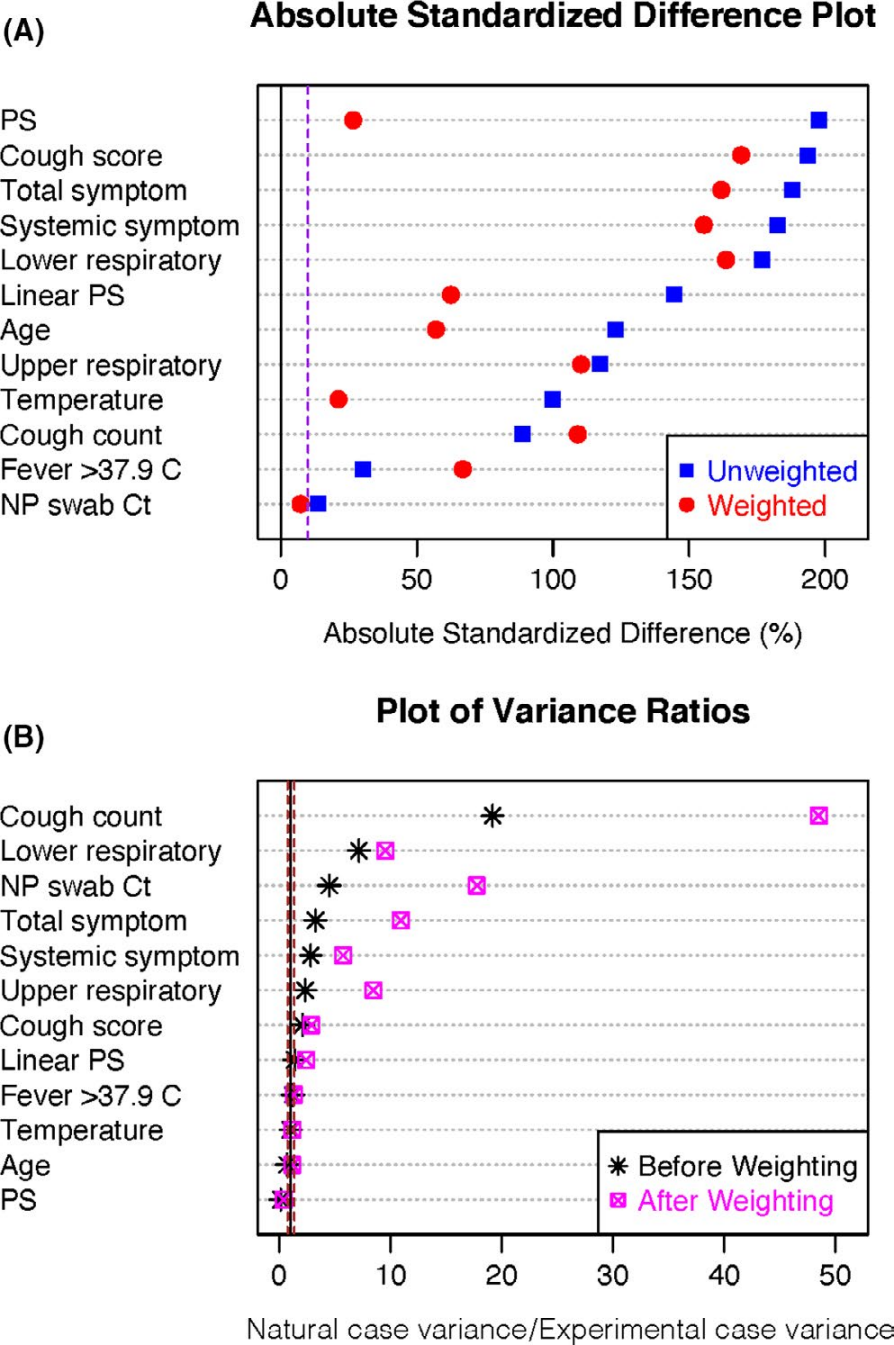
Balance diagnostics for propensity score adjustment by average treatment effect, where the naturally infected cases are “treatment.” A, Absolute standardized differences between experimental and naturally infected case covariates plotted for each covariate, the propensity score (PS) and the linear PS. The dotted line represents 10%, which balanced groups do not generally exceed. B, Variance ratios. Dotted lines represent 0.5 and 2, the range for which balanced populations generally do not exceed

After extensive testing, the best propensity score model (covariates: fever >37.8°C, body temperature, and upper respiratory symptom score) and adjustment by inverse probability weighting for average treatment effect (ATE) minimized the standardized differences between the groups with mean absolute value standardized difference of 91 (Table S5). After ATE adjustment, balance improved for some covariates, however not to the point where they could be considered similar (Figure 3, Table S5). Although it is advisable that absolute standardized differences for covariates not exceed 10% between comparison groups, and variance ratios approach one (range 0.5‐2),^17^ the best model with weighted adjustment had absolute standardized differences ranging 7.3%‐169.1% and variance ratios up to 48.5. The substantial differences between covariate distributions did not support the use of propensity score–adjusted approaches for making further comparisons between these populations.

## 4 DISCUSSION

We compared aerosol RNA shedding in influenza A (H3) cases infected naturally and by nasal instillation under experimental conditions. Previously, we cultured influenza virus from exhaled breath showing that quantitative culture correlates with RNA copy detection (*r* = .34, *P* < .0001).^15^ A minority of experimental cases shed virus into aerosols (28%). A far greater proportion of the naturally infected study population shed into aerosols (86%). Among the experimentally infected with detectable viral aerosols, the fine RNA copy GM was within log_10_ that for naturally infected cases. A more substantial difference in fine aerosol shedding rate was observed at the level of the overall distribution, with increases in median and upper percentiles for naturally infected cases (Figure S4). Compared with naturally infected cases at the 95th percentile of fine aerosol shedding, experimentally infected cases shed nearly 2.5 log_10_ fewer RNA copies. Given the selection of naturally infected cases on ILI symptoms and/or a positive rapid antigen test, and an observed relationship between symptoms and shedding (Table 3), it is possible that aerosol shedding observed for the naturally infected study populations overestimates what might be expected for a sample representative of all naturally acquired infections.

Peak RNA copies shed into aerosols on day 3 post‐nasal inoculation was one day later than that observed in upper respiratory mucosa in previous challenge studies,^7^ yet consistent with the 1‐2 day post‐symptom onset nose and throat swab viral load peak from household contact surveillance studies.^8^ There are no available data with which to compare the current study's aerosol shedding. If we take previous estimates of ~ 1‐2 days as the influenza A incubation period, plus about day 1 post‐symptom onset to reach peak aerosol shedding in the naturally infected population, then we would expect peak aerosol shedding on ~2‐3 days following exposure to virus. This is consistent with the peak in aerosol shedding in the experimentally infected cases at day 3 post‐inoculation, suggesting that the progression of infection from exposure to replication is consistent with the natural infection group and with other studies. The significant decrease in aerosol shedding by day post‐onset (Table 3) among the naturally infected, but not the experimental cases, could be related to failure to detect a clear trend against a background of very much lower overall aerosol shedding for the experimental group. The temporal decline observed for naturally infected cases along with previous reports of less temporal decline in viral load from nasopharyngeal swabs compared with fine aerosols following day 1 post‐symptom onset,^15^ and a tendency for nasal viral load to overestimate transmission risk after day 3 post‐symptom onset,^12^ suggests aerosol shedding may better fit epidemiologically observed transmission dynamics over time in the household setting.

Unadjusted lower respiratory and cough symptom scores were associated with viral aerosol detection in both groups with a clear dose‐response relationship in cough score for the naturally infected cases, supporting the notion of a symptomatology‐shedding relationship for aerosols. Peak symptom scores and aerosol shedding coincided, consistent with the temporal dynamics of other studies.^7^, ^8^, ^9^ However, regression analyses that restricted observations to maximum fine aerosol samples found mostly weak and unstable effects of symptoms on shedding rate (Table S4). These data should be interpreted with caution given limited heterogeneity in symptom severity, with milder illness characteristic of the experimental cases and more moderate to severe illness characteristic of the naturally infected cases. It may be for this reason that other studies have reported mixed results with respect to associations between symptomatology and nose/throat viral load.^9^, ^10^ Nevertheless, that fever was observed in 43% of naturally infected and 15% of experimentally infected cases and may indicate that febrile periods of illness are associated with increased infectivity risk. Studying cases with a broader range of symptom severity could provide additional insight into symptom‐shedding relationships with useful implications for identifying contagious symptom profiles.

There is growing evidence that airborne transmission plays an important role in the spread of influenza.^5^, ^14^, ^18^, ^19^ Humans experimentally challenged to influenza virus by airborne particles had a 50% risk of infection to a 0.6‐3.5 TCID_50_ dose and exhibited increased propensity for moderate to severe illness with fever and cough compared with others experimentally challenged by nasal droplets.^1^, ^2^ The term anisotropic has been used to describe such infections where inoculation mode determines illness presentation.^4^, ^5^ A population of cases with naturally acquired infections may be expected to demonstrate a higher proportion of moderate‐severe influenza‐like illness compared with a population of cases exclusively infected by exposure to the nasal mucosa. Compared with other symptoms, systemic scores declined more rapidly following day 1 post‐symptom onset for natural cases, consistent with other findings^8^ and suggestive of the immune system clearing systemic infection. These findings may hint that natural cases may be more likely to result in lung and systemic infection initiated by an airborne dose, whereas experimentally infected cases with only nasal mucosal exposure were more likely to have few if any systemic symptoms and illness more localized to the upper respiratory tract.

Given the selection bias and the contrasted symptom profiles in two populations and observed associations between symptoms and shedding, we attempted to adjust for the effect of symptoms to understand the direct effect of experimental vs natural infection on the viral load in fine particle aerosols (Figure 3, Figure S1 of SI Appendix S2). Ultimately, propensity score modeling failed to balance the distribution of covariates between groups and we concluded that the groups were simply too different to achieve an unbiased estimate of the main effect of group membership (experimental vs natural) on shedding strength. Assuming minimal contribution of potential confounders on the pathway between mode of inoculation and study population membership (ie, age, sex, host immunity, virus pathogenicity, dose), we cannot conclude that the unadjusted differences in symptomatology and shedding are a result of mode of inoculation, or simply the result of the differences inherent in recruitment and enrollment procedures and other potentially unobserved confounders in the absence of a randomized controlled design. Because it is unclear that any subsets of cases from each group could be selected to make valid comparisons, we decided to present crude population differences with respect to viral aerosol shedding in Tables 2, 3, and Table S4, and include everyone meeting case definition (including the 5/39 asymptomatic volunteer cases). We emphasize that all cases had qRT‐PCR evidence of infection (Methods).

Identifying naturally infected reference groups that represent the true distribution of symptom severity presents a challenge. Although a substantial proportion of cases are asymptomatic, symptomatic community cases are more prone for inclusion in epidemiologic studies upon seeking medical attention.^20^ Multi‐year sero‐surveillance of large cohorts in the UK shows influenza infections presented asymptomatically at a rate of 77 per 100 person‐seasons.^21^ A meta‐analysis of longitudinal studies using serological evidence of infection and controlling for background illness reported a 65%‐85% asymptomatic fraction.^22^ It is possible that the experimentally infected cases are not substantially different in symptomatology from a representative sample of all influenza infections. Accessing a representative sample might be achieved through intensive household or dormitory surveillance of contacts of known cases.

The infectious dose for airborne influenza and the infectious potential of cases infected by various modes are largely unknown. If the typical fine aerosol shedding rate from influenza cases is important for driving airborne transmission, then our findings would indicate that nasal mucosal exposure in the experimental challenge model produces cases with airborne infectious potential similar to symptomatic cases infected naturally by contact, large droplets, or fine aerosols. If above‐average shedders are important for driving airborne transmission (ie, superspreader hypothesis), then infections acquired through nasal mucosa may not pose as much airborne infectious potential. If we assume hypothetically that the symptomatic naturally infected cases drawn from the University of Maryland campus community represent the upper 1% of symptom severity and shedding strength in a broader population, and if we also assume that the experimental cases are representative of total community infections, the chances of an experimental case reaching the level of fine aerosol shedding observed in the naturally infected group would be 0.39% (1% of 39 experimental cases). If shedders in the upper percentiles of shedding rate are responsible for driving transmission, then it would take many more experimental cases to adequately simulate transmission events in a human transmission challenge trial model. This introduces logistical challenges and motivates work to identify, among naturally infected shedders, characteristics predictive of aerosols (and mucosal) shedding. In particular, response to infection by different modes may vary between children and adults, with implications for subsequent infectivity and population epidemiology.^10^, ^13^, ^23^, ^24^ Clinical detection of infections that may be associated with disease severity and potential for self‐isolation or other behaviors that could modify contagiousness should be considered in population‐level transmission risk assessment.

Given bias introduced by selection of natural infections on symptoms plus fever or rapid antigen test, the observed correlations of symptoms and fever with viral shedding into aerosols, and a small N with minimal covariable distributional overlap precluding appropriate adjustment, we conclude that the naturally infected population is too different from the experimentally infected cases to make valid comparisons. Our observations show that the 52 nasally inoculated experimental cases produced viral aerosol shedders less frequently than the 83 symptomatic naturally infected population. When they did shed detectable virus into aerosols, the experimental cases did so at substantially lower quantities than the symptomatic naturally infected group. This difference in aerosol shedding was most pronounced when comparing the highest percentiles of aerosol shedding for each group. The probability and quantity of aerosol shedding in unselected natural infection is unknown. Therefore, these findings encourage efforts to evaluate shedding from infections observed during contact surveillance without selection based on symptoms or fever.

## CONFLICT OF INTEREST

JSN‐V‐T and BK declare previous consultancy fees from hVIVO plc, unrelated to the current work. JSN‐V‐T is currently seconded to the Department of Health and Social Care (DHSC), England; the views expressed in this paper are not necessarily those of DHSC. RLW, AG, JF, and AM are/were employees of hVIVO plc each of whom hold shares and/or share options in the company.

## AUTHOR CONTRIBUTION

**Paul Jacob Bueno de Mesquita:** Conceptualization (supporting); Data curation (lead); Formal analysis (lead); Investigation (equal); Methodology (equal); Software (lead); Visualization (lead); Writing‐original draft (lead); Writing‐review & editing (lead). **Jonathan Nguyen‐Van‐Tam:** Conceptualization (equal); Data curation (equal); Formal analysis (supporting); Funding acquisition (lead); Investigation (lead); Methodology (equal); Project administration (lead); Resources (lead); Supervision (equal); Writing‐review & editing (equal). **Ben Killingley:** Conceptualization (equal); Data curation (equal); Formal analysis (supporting); Funding acquisition (supporting); Investigation (equal); Methodology (equal); Project administration (equal); Writing‐review & editing (equal). **Joanne Enstone:** Conceptualization (equal); Data curation (equal); Formal analysis (supporting); Funding acquisition (supporting); Investigation (equal); Methodology (supporting); Writing‐review & editing (equal). **Robert Lambkin‐Williams:** Formal analysis (supporting); Investigation (equal); Methodology (supporting); Writing‐review & editing (equal). **Anthony S. Gilbert:** Formal analysis (supporting); Investigation (equal); Methodology (supporting); Writing‐review & editing (equal). **Alexander Mann:** Formal analysis (supporting); Investigation (equal); Methodology (supporting); Writing‐review & editing (equal). **John Forni:** Formal analysis (supporting); Investigation (equal); Methodology (supporting); Writing‐review & editing (supporting). **Jing Yan:** Data curation (supporting); Formal analysis (supporting); Writing‐review & editing (supporting). **Jovan Pantelic:** Data curation (supporting); Formal analysis (supporting); Investigation (equal); Methodology (equal); Writing‐review & editing (equal). **Michael L. Grantham:** Data curation (supporting); Formal analysis (supporting); Investigation (equal); Methodology (equal); Writing‐review & editing (equal). **Donald K. Milton:** Conceptualization (equal); Data curation (supporting); Formal analysis (equal); Funding acquisition (equal); Investigation (equal); Methodology (equal); Project administration (equal); Resources (equal); Supervision (equal); Validation (equal); Writing‐review & editing (equal).

## Supporting information

Supplementary Material
