# Supplementary material for: Influenza A (H3) illness and viral aerosol shedding from symptomatic naturally infected and experimentally infected cases
Source: Influenza Other Respir Viruses. 2020 Jul 23;15(1):154–63. doi: 10.1111/irv.12790 (PMC7767952; doi:10.1111/irv.12790)
Supplement: Supplementary file 1 — Supplementary Material [file IRV-15-154-s001.docx]

**SUPPLEMENTARY INFORMATION (SI)**

SI for:

Influenza A (H3) illness and viral aerosol shedding from symptomatic naturally infected and experimentally infected cases

Corresponding authors:

Paul Jacob Bueno de Mesquita, PhD (jbueno@umd.edu) and Donald K. Milton, DrPH, MD (dmilton@umd.edu)

**Contents:**

- EMIT Consortium Team Members
- Appendix 1: Selection of naturally infected cases for analysis from larger population of naturally infected community cases collected at University of Maryland
  - Table S1
- Appendix 2: Description of comparison analysis and profile of studies that generated data
  - Figure S1
- Appendix 3: Propensity score workflow
  - Table S2
- Figure S2
- Appendix 4. Cough count imputation
- Appendix 5. Age and sex differences between experimentally and naturally infected cases
- Table S3-S6
- Figures S3-S4
- SI References

**EMIT Consortium Team Members**

EMIT team members were: Walt Adamson, Blanca Beato-Arribas, Werner Bischoff, William Booth, Simon Cauchemez, Sheryl Ehrman, Joanne Enstone, Neil Ferguson, John Forni, Anthony Gilbert, Michael Grantham, Lisa Grohskopf, Andrew Hayward, Michael Hewitt, Ashley Kang, Ben Killingley, Robert Lambkin-Williams, Alex Mann, Donald Milton, Jonathan Nguyen-Van-Tam, Catherine Noakes, John Oxford, Massimo Palmarini, Jovan Pantelic, and Jennifer Wang. The Scientific Advisory Board members were: Allan Bennett, Ben Cowling, Arnold Monto, and Raymond Tellier.

**Appendix 1. Selection of naturally infected cases for analysis from larger population of naturally infected community cases collected at University of Maryland**

The set of data from the community cases observed in the University of Maryland campus community are different from that of the “complete data” used in the analyses of Yan et al., 2018.^1^ The data in the current manuscript come from the set of 158 qRT-PCR confirmed influenza cases with exclusion of all breath collection visits from four dual influenza infections, breath collection visits on days 0 and >3 post symptom onset, those with incomplete qRT-PCR data, and those that were not H3 infections confirmed by qRT-PCR of nasopharyngeal swab. Cough counts from three exhaled breath sampling visits that were missing and thus excluded by Yan and colleagues, were imputed for this analysis. Table S1 provides a summary of cases and exhaled breath collection visits selected for analysis from 178 enrolled community cases.

**Table S1. Community case exclusion and inclusion to achieve analytical dataset**

|  | **Exclusions** | | | | | **Inclusions** | |
| --- | --- | --- | --- | --- | --- | --- | --- |
| **Reason for exclusion** | **Number subjects with all breath collection excluded** | **Number breath collection visits excluded from subjects with all breath collection visits excluded** | **Number subjects with at least 1 but not all breath collection visits excluded** | **Number breath collection visits excluded from subjects with at least 1 but not all breath collection visits excluded** | **Total breath collection visits excluded**  **(sum of the 3^rd^ and 5^th^ columns)** | **Running count of subjects** | **Running count of breath collection visits** |
| **Total screened** | - | - | - | - | - | 355 | 276 |
| **Case inclusion criteria^†^** | 177 | 0 | 0 | 0 | 0 | 178 | 276 |
| **Not confirmed by qRT-PCR** | 20 | 26 | 0 | 0 | 26 | 158 | 250 |
| **Day 0, >3 post symptom onset** | 9 | 8 | 9 | 10 | 18 | 149 | 232 |
| **Incomplete qRT-PCR data^‡^** | 5 | 6 | 1 | 1 | 7 | 144 | 225 |
| **Dual infection^§^** | 3 | 4 | 0 | 0 | 4 | 141 | 221 |
| **Non H3 infection** | 58 | 75 | 0 | 0 | 75 | 83 | 146 |
| **Overall** | **95** | **119** | **10** | **11** | **130** | **83** | **146** |
| **^†^** Positive Quidel QuickVue rapid influenza test, or oral temperature >37.8 °C, plus cough or sore throat, and (ii) presented within the first 3 d of symptom onset.  **^‡^** Description of the incomplete PCR data:   - Drop subject 333 1 and only G-II visit because lost coarse aerosol sample (although good data exists for the NP and the fine aerosol) - Drop subject 52 because false positive (this makes 52 a negative case and thus we exclude all sampling instances) - Drop subject 58 because false positive (58 only had 1 G-II sampling instance so this was excluded) - Drop subject 182 2nd G-II visit because bad inter-run calibrator on the PCR (there is still a 1st G-II visit for 182 so this subject is not excluded entirely) - Drop subject 322 because bad inter-run calibrator on the PCR (this was the only G-II sampling instance, so this subject is excluded entirely) - Drop subject 337 because bad inter-run calibrator on the PCR (this was the only G-II sampling instance, so this subject is excluded entirely)   **^§^** Dual infections:   - 1 instance: H3N2 and Pandemic H1 - 1 instance: B and unsubtypable A - 1 instance: B and H3N2 | | | | | | | |

**Appendix 2. Description of comparison analysis and profile of studies that generated data**

Figure S1 shows the enrollment profiles of the two studies and DAG (directed acyclic graph) used for making comparisons between experimentally and naturally infected influenza A/H3 cases. The main outcomes of interest are the probability of shedding virus into aerosols and the rate of viral shedding in aerosols. Given their role in transmission, fine particle aerosols are of particular interest. Other comparisons of interest are the temporal dynamics of illness, the temporal dynamics of aerosol shedding, and the overlap of symptoms with strength and duration of shedding.

This analysis focuses on the shedding and symptomatology differences between the population of symptomatic naturally infected influenza A/H3 cases and the influenza A/H3 cases infected under experimental conditions (Figure S1). Ultimately, the main independent variable of interest in this line of research is the mode of inoculation, with the experimentally challenged infections representing contact transmission via exposure to nasal mucosa, and the naturally infected cases representing transmission by any plausible mode (the red rectangle in the DAG). Other differences may exist in the two study populations related to the conditions of infection initiation including systematic differences in age, sex, immunity, and dose. Although these potential confounders may not be completely controlled for by study design, they are not likely to play a major role in confounding the interpretation that experimentally infected cases represent contact or large droplet spray transmission to the upper respiratory tract and naturally infected cases represent transmission by any plausible mode. Brief discussion follows about each of these potential confounders.

The source population for the observational study and the inclusion criteria for the challenge study favored recruitment of healthy, young adults. The age of the young adults was several years higher but unlikely to drive differences in susceptibility. Susceptibility is likely to be higher for young children or elderly populations. Male and female were well balanced in study population of naturally infected cases; however, this distribution was skewed in the trial study with a majority male infections. Epidemiological evidence from various influenza seasons, and illnesses in various age groups suggests that males may have elevated risk hospitalization, suggesting a potential role for sex in influenza pathogenesis.^2^ It is uncertain to what extent sex may influence susceptibility to infection in these study populations, but it is acknowledged as a potential confounder in the relationship between study population membership and infection initiation for members of the studies. Virally challenged volunteers were screened for low levels of pre-existing antibodies (HAI ≤10), while the naturally infected population was not. The symptomatic, naturally acquired infections were likely to have had less immunity compared with the total source population because they were infected. But, a small subset of naturally infected cases had been immunized for influenza for the current and/or previous influenza seasons, pointing toward a range of pre-existing antibodies above the exclusion threshold of the trial study.^1^ Thus, pre-existing antibodies were unmeasured in the naturally infected study population and could be a confounding factor related to the infection conditions between the study populations. The laboratory prepared virus used for human challenge could have been attenuated compared with wildtype, but virologic investigation of this virus suggests that the HA variant matches other wildtype viruses and may not be a major source of difference.^3,4^ Finally, the dose leading to infection may have been different between the two study populations and could also be a confounding variable. Volunteers received an intranasal dose of 5.5 log_10_ TCID_50_, however the dose leading to infection in the naturally infected populations was unmeasured. It has been shown that low doses of aerosolized virus can initiate infection.^5^ In naturally infected cases not infected by aerosols (i.e., infected similarly to infected volunteers, via contact exposure to upper airway mucosa), they may have been exposed to a range of doses and correlations between dose and viral shedding peak and duration have been reported in volunteers challenged with influenza virus.^6^ Overall, potential confounding factors between transmission mode – the main independent variable of interest – and the initiation of infection that may not have been fully controlled for in the designs of the experimental and observational studies were age, sex, host immunity, viral pathogenicity, and dose. Assessment of these potential confounders leads us to believe that they are not likely to have a substantial effect in obscuring the main effect of mode on the conditions of infection in the study populations.

Covariates are illustrated as confounders because there is reported evidence and/or biological plausibility for considering them so based on their expected effects on the independent variable (study population membership) and the dependent variables (aerosol shedding probability and rate). We attempted to achieve acceptable balance across these confounders with propensity score model adjustment, however, were unable to do so because the populations were simply too different in their distributions for these variables.


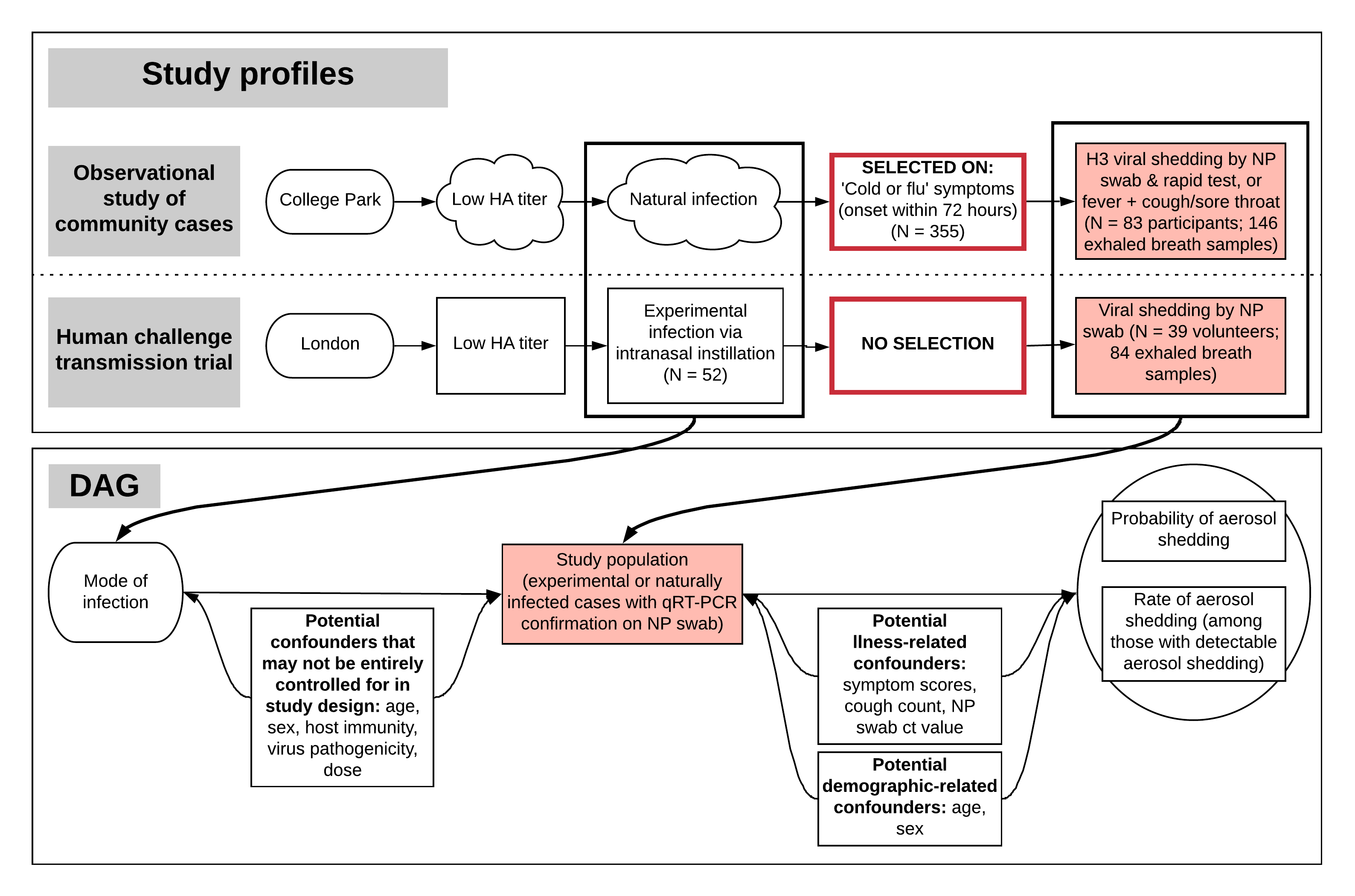


**Figure S1.** Profiles of the EMIT studies and directed acyclic graph (DAG). (Top) study profiles used to make comparisons between experimental and naturally infected populations. Cloud shapes depict unobserved variables that are believed to be true. DAG (bottom) for comparing aerosol shedding in the study populations.

**Appendix 3. Propensity score workflow**

**Table S2. Codebook of the data frame used in propensity score analyses**

| **Variable** | **Type of Variable** | **Description** |
| --- | --- | --- |
| subject.id | Subject ID # (numeric, integer variable) | ID numbers below 400 are UMD Flu Cases. ID numbers above 400 are Infected Donors. |
| Group | Factor with two levels, 0 and 1 (the “Group” variable is actually numeric and uses 0 and 1 as the only possible options: 0 for Infected donors and 1 for UMD flu cases; but the “Group_factor” variable is the factor variable and uses the text “Infected donors” and “UMD Flu Cases” as the levels. | Coded as 0 = Infected donors; 1 = UMD flu cases |
| Group_factor | Factor with two levels 0 and 1 | Coded as 0 = Infected donors; 1 = UMD flu cases (the levels of the variable are written as “Infected donors” and “UMD Flu Cases” |
| Age | Numeric, integer variable | Age in years |
| Sex | Factor with two levels, 0 and 1 (although technically this “sex” variable is coded as numeric with possible levels 0 and 1 and the sex_factor variable is the one that is factor with levels “female” and “male”) | 0 = female, 1 = male |
| Sex_factor | Factor with two levels | “female” and “male” (with female coded as 0 and male as 1 in the “sex” variable) |
| Upper_resp | Numeric, discrete | Sum of the ordinal (4-level from 0-3 with 0=none and 3=severe symptom) for the following symptoms: runny nose, stuffy nose, sneezing, sore throat, and earache, score range 0–15. |
| Lower_resp | Numeric, discrete | Sum of the ordinal (4-level from 0-3 with 0=none and 3=severe symptom) for the following symptoms: shortness of breath, and cough, score range 0–6 |
| Systemic_sx | Numeric, discrete | Sum of the ordinal (4-level from 0-3 with 0=none and 3=severe symptom) for the following symptoms: malaise, headache, muscle/joint  ache, score range 0–9 |
| Total_sx | Numeric, discrete | Sum of the ordinal (4-level from 0-3 with 0=none and 3=severe symptom) for the following symptoms: runny nose, stuffy nose, sneezing, sore throat, earache, shortness of breath, cough, malaise, headache, muscle/joint  ache: score range 0–30. |
| Cough | Numeric, discrete (factor with 4 levels) | 4 levels for symptoms severity with 0 as no symptom to 3 as most severe symptom |
| Cough_factor | Factor, 4 levels | "0-none", "1-mild", "2-moderate", "3-severe" coded for cough self-reported symptom scores of 0, 1, 2, and 3, respectively. |
| cough_count_full | Numeric, discrete. | Number of times coughed during 30-minute sample collection in the g-ii machine. Included 2 instances of missing cough in the Infected donor group |
| Body_temp | Continuous (1 decimal place) |  |
| Ever_fever | Numeric, discrete (factor with 2 levels) | 1 = had fever > 37.9 degrees C on any of the days of observations (1-4 days post inoculation for donors and 1-3 day post symptom onset or during University Health Center visit for UMD Flu Cases). 0 = temperature reading was below the study threshold for “febrile illness” |
| Ever_febrile_factor | Factor (2 levels) | Coded as “yes” = 1 (yes, was febrile at some point during observation), “no” = 0 (no, was never observed to reach the body temperature threshold for febrile). |
| NP_ct | Continuous (2 decimal places) | CT value on the qRT-PCR assay for influenza A viral RNA. |
| sample_mean_copies | Continuous outcome | RNA copies – the mean of replicates for samples with detectable virus (have not used tobit to impute for samples where there was not at least 1 replicate above detection limit). |
| **Variables in df but not used** | | |
| study.day | Character variable with 4 levels. | The 4 levels represent the possible study days. For UMD Flu Cases the study day refers to the three days post symptom onset and for Infected donors the study day refers to the four days post inoculation. |
| sample.type | Character variable with 2 possible levels | For this df this variable is always “Condensate” because the fineflu data frame has been filtered to included only the condensate samples and their associated clinical evaluation data. |
| Date | Character | Date of the data collection for the associated observation. |
| Nose_run | Numeric, discrete (factor with 4 levels) | 4 levels for symptoms severity with 0 as no symptom to 3 as most severe |
| Nose_stuf | Numeric, discrete (factor with 4 levels) | 4 levels for symptoms severity with 0 as no symptom to 3 as most severe |
| Sneeze | Numeric, discrete (factor with 4 levels) | 4 levels for symptoms severity with 0 as no symptom to 3 as most severe |
| Throat_sr | Numeric, discrete (factor with 4 levels) | 4 levels for symptoms severity with 0 as no symptom to 3 as most severe |
| Earache | Numeric, discrete (factor with 4 levels) | 4 levels for symptoms severity with 0 as no symptom to 3 as most severe |
| Malaise | Numeric, discrete (factor with 4 levels) | 4 levels for symptoms severity with 0 as no symptom to 3 as most severe |
| Sob | Numeric, discrete (factor with 4 levels) | 4 levels for symptoms severity with 0 as no symptom to 3 as most severe |
| Headache | Numeric, discrete (factor with 4 levels) | 4 levels for symptoms severity with 0 as no symptom to 3 as most severe |
| Mj_ache | Numeric, discrete (factor with 4 levels) | 4 levels for symptoms severity with 0 as no symptom to 3 as most severe |
| Febrile_day | Numeric, discrete (factor with 2 levels) | 1 = had fever > 37.9 degrees C on the associated day of data collection. 0 = temperature reading was below the study threshold for “febrile illness” |
| Cough_number | Numeric, discrete. | Number of times coughed during 30-minute sample collection in the g-ii machine. Included 2 instances of missing cough in the Infected donor group |
| Restricted to the observation from the day with the maximum fine aerosol shedding for each participant (N=11 for experimentally infected EMIT viral donors and N=71 observations for naturally infected UMD Flu Cases). | | |

**Specifying propensity score model with continuous aerosol shedding outcome.** The propensity score model is a logistic regression model. It regresses the logit of the study population (natural versus experimental infection) on a set of covariates. Comparison groups conditioned on an appropriately specified propensity score should have similar means and variances, with standardized differences of close to 0 and variance ratios of close to 1.^7^ Propensity score models that achieved comparison group balance after conditioning on propensity score were considered for use in estimating the effect of comparison group membership on the outcomes of interest. Propensity score model specifications that achieved balanced population means were also tested against Donald Rubin’s propensity score balancing criteria. Rubin’s criteria assesses the degree of overlap between propensity scores in each group. Correctly specified propensity score models that also have enough overlap in propensity scores sufficiently minimize bias introduced by lack of group randomization, with the acknowledgement that unmeasured confounders may still introduce bias. None of the 14 propensity score models tested achieved enough covariate balance between naturally and experimentally infected study populations, even after truncating the sampling frame from both groups. Thus, we conclude that the populations are too different to make support valid comparisons between groups with respect to the outcome, even if covariate adjustment should be used.

In the initial propensity score model, predictors of experimental or natural infection group were selected on the basis of their relationship with the outcome of observed shedding into fine aerosols above the detection limit. The lower respiratory symptom score, cough symptom score, and the cough count observed during half-hour exhaled breath collection all had positive, statistically significant effects on the odds of viral shedding into fine aerosols. Since the cough symptom score makes up 50% of the lower respiratory score and likely drives the effect seen in the lower respiratory score, the cough score and not the lower respiratory symptom score was included. Thus, the initial propensity score model included only cough symptom score and observed cough count as predictors. Overlap in propensity scores between study population groups decreased when including lower respiratory scores, affirming this decision.

Propensity score model specifications need not be concerned with multicollinearity because the estimated propensity scores and not the variances are of main interest. Age was associated with study population, however published literature assessing propensity score model specification have warned against including variables that are associated with group membership but not the outcome of interest because of the risk of increasing variance in the estimated effect without reducing bias.^8,9^

Donald Rubin’s rules for assessing propensity score balance were used to evaluate propensity score model specifications.^10^ Failure of balance between covariates in the two study populations indicates the need for adjustment using three propensity score balancing methods: matching, subclassification, inverse probability weighting. Valid of the effect of natural infection compared with experimental infection, in our specific studies, can be carried out after improving balance to an acceptable range.

- First rule: The absolute value of the standardized difference of the linear propensity score should be close to 0 and definitely less than 50.
- Second rule: The ratio of variances of the linear propensity score between the comparison groups (i.e., natural to experimental infection). This should be close to 1 and definitely between ½ and 2.
- Third rule: After regressing the residuals for each covariate specified in the propensity score model on the linear propensity score, the ratio of variance of residuals between comparison groups (i.e., natural to experimental infection) close to 1.

**Figure S2.** Mean symptom scores and observed cough counts over time (N=84 experimental and N=146 natural observations). Bars represent standard error around the mean. The comparison of symptomatology includes data from the entire study population for each group (including observations from 5/39 asymptomatic experimentally infected volunteers) collected at time of exhaled breath sampling.

**Appendix 4. Cough count imputation**

Cough count data was imputed in the missing five (6.0%) and three (2.1%) exhaled breath collection instances for experimental and natural cases, respectively. For experimental cases, a linear mixed model with fixed effects of cough symptom score, study day, and sex, with a random effect of subject was used to predict cough count. To impute missing cough counts in the naturally infected group, we first used Tobit regression with fixed effect of study day and random effect of person to estimate fine aerosol shedding values for all observations. Next, to estimate missing cough counts, we used a linear mixed model with fixed effects of cough symptom score, estimated fine aerosol RNA shedding, sex, age, BMI, and day post-symptom onset, with random effect of subject. Available variables related to cough^1^ were tested in models with those that minimized AIC selected.

**Appendix 5. Age and sex differences between experimentally and naturally infected cases**

Age was not associated with probability or strength of fine aerosol shedding in either study population (Tables S1), which is expected given the makeup of healthy, young adults. Only males shed into aerosols in the experimentally infected group. This finding might be explained by the low probability of shedding into aerosols (28%) compounded with the low number of females in the study (N=11). This difference may weaken the generalizability of the challenge model used in EMIT. There is preliminary evidence from studies of vaccine-associated immunity that TLR7 is involved in adaptive immune response to influenza virus through B cell activation. This could provide women with an immune system advantage given TLR7 encoding on the X-chromosome and incomplete inactivation of the second allele.^12,13^ The uncertainty surrounding sex-related immunology against influenza infection warrants more work.

**Table S3. Covariates in experimental and naturally infected cases from fine aerosol shedders on day of maximum shedding**

| **Variables** | **Experimental** | **Natural** | **Standardized Difference %** | **P value**  **(parametric)^†^** | **P value**  **(non-parametric)^‡^** |
| --- | --- | --- | --- | --- | --- |
| **Age** | 31.09 (8.01) | 21.82 (6.70) | -123.0 | <0.001 | <0.001 |
| **Participants with fever >37.9^o^C (%)** | 3.00 (27.3) | 30.03 (42.3) | 30.3 | 0.352 | 0.352 |
| **Temperature (^o^C)** | 36.76 (0.66) | 37.46 (0.67) | 99.9 | 0.001 | <0.001 |
| **Upper respiratory score** | 3.64 (1.80) | 7.01 (2.75) | 117.4 | <0.001 | 0.002 |
| **Lower respiratory score** | 0.46 (0.52) | 3.37 (1.40) | 176.7 | <0.001 | <0.001 |
| **Systemic symptom score** | 1.18 (1.25) | 5.86 (2.10) | 182.4 | <0.001 | <0.001 |
| **Total symptom score** | 5.27 (2.61) | 16.24 (4.70) | 187.8 | <0.001 | <0.001 |
| **Cough score** | 0.46 (0.52) | 2.32 (0.75) | 193.6 | <0.001 | <0.001 |
| **Cough count** | 4.00 (6.42) | 28.48 (28.10) | 88.9 | <0.001 | 0.002 |
| **Nasopharyngeal swab Ct value** | 23.18 (2.84) | 22.40 (6.02) | -13.6 | 0.479 | 0.048 |
| **Propensity score** | 0.48 (0.34) | 0.93 (0.14) | 197.6 | <0.001 | <0.001 |
| **Linear propensity score** | -0.30 (2.54) | 4.53 (2.97) | 144.4 | <0.001 | <0.001 |
| Reporting mean (standard deviation) for each variable except for ever fever, from N=11 experimental and N=71 naturally infected cases (selected during their maximal aerosol shedding day). The mean and max absolute value standardized differences were 122.0% and 197.6%, respectively. Kolmogorov-Smirnov test for the mean standardized difference was 0.686 (p<0.001). Sex was removed because no females shed into fine aerosols in the experimental group.**^†^**t-tests. **^‡^**Kolmogorov-Smirnov tests. | | | | | |

**Table S4. Unadjusted changes in RNA copies shed into fine aerosols**

| **Predictor** | **Experimental**  **Estimate (CI)** | **Natural**  **Estimate (CI)** |
| --- | --- | --- |
| **Age** | 4.20E+2  (-2.59E+3, 3.43E+3) | 5.62E+4  (-1.80E+5, 2.93E+5) |
| **Sex** | - | 8.19E+5  (-2.33E+6, 3.97E+6) |
| **Ever fever >37.9^o^C** | 1.00E+4  (-4.13E+4, 6.13E+4) | 1.39e+6  (-1.78e+6, 4.57e+6) |
| **Body temperature (^o^C)** | 1.39E+4  (-2.12E+4, 4.90E+4) | 2.21E+6  (-1.14E+5, 4.53E+6) |
| **Upper respiratory score** | -1.72E+3  (-1.51E+4, 1.16E+4) | -1.26E+5  (-7.03E+5, 4.50E+5) |
| **Lower respiratory score** | 1.84E+4  (-2.58E+4, 6.26E+4) | -1.32E+5  (-1.27E+6, 1.00E+6) |
| **Systemic symptom score** | -6.40E+3  (-2.51E+4, 1.24E+4) | -4.03E+5  (-1.15E+6, 3.46E+5) |
| **Total symptom score** | -1.55E+3  (-1.07E+4, 7.64E+3) | -1.35E+5  (-4.71E+5, 2.01E+5) |
| **Cough symptom score ^‡^** | | |
| No cough symptom | 1.00 (REF) | 1.00 (REF) |
| Mild | 1.84E+4  (-2.58E+4, 6.26E+4) | 1.02E+6  (-1.30E+7, 1.50E+7) |
| Moderate | - | 9.92E+5  (-1.25E+7, 1.45E+7) |
| Severe | - | 3.34E+6  (-1.01E+7, 1.68E+7) |
| **Cough count** | 1.42E+4  (-5.74E+3, 3.41E+4) | 6.78E+5  (-1.12E+6, 2.48E+6) |
| **Nasopharyngeal swab Ct** | -3.39E+3  (-1.15E+4, 4.75E+3) | -1.32E+5  (-3.94E+5, 1.30E+5) |
| Observations from only the maximum fine aerosol shedding day for each subject (N=11 experimental, N=71 natural). Change from a single unit increase in age, body temperature, symptoms scores, an nasopharyngeal swab Ct, an IQR increase in cough count, ever fever >37.9^o^C versus afebrile, male versus female. Bold: significant at p=0.05. **^†^**Only males shed into aerosols in the experimental group. **^‡^**Cough scores of 2 or 3 were never observed in the experimental group. | | |

**Table S5 (part 1/3). Quality of covariate balance after propensity score adjustment**

|  | **Model^†^ 1** | | **Model 2** | | **Model 3** | | **Model 4** | | **Model 5** | |
| --- | --- | --- | --- | --- | --- | --- | --- | --- | --- | --- |
| **Approach** | **Standardized differences** | **Variance ratios** | **Standardized differences** | **Variance ratios** | **Standardized differences** | **Variance ratios** | **Standardized differences** | **Variance ratios** | **Standardized differences** | **Variance ratios** |
| **No adjustment** | (-125.6, 288.9) | (0.7, 19.2) | (-125.6, 288.9) | (0.7, 19.2) | (-125.6, 288.9) | (0.7, 19.2) | (-125.6, 288.9) | (0.7, 19.2) | (-125.6, 288.9) | (0.7, 19.2) |
| **1:1 Propensity Matching^‡^** | (-305.3, 130.6) | (0.4, 8.0) | (-312.6, 129.5) | (0.1, 8.2) | (-305.3, 130.6) | (0.4, 8.0) | (-312.6, 127.2) | (0.2, 9.6) | (-312.6, 129.5) | (0.1, 8.2) |
| **Propensity subclassification by stratification^§^** | | | | | | | | | |  |
| Sextile 1 | (-128.2, 425.6) | (0.0, 6.4) | (-128.2, 425.6) | (0.0, 6.4) | (-138.2, 425.6) | (0.0, 6.4) | (-128.2, 425.6) | (0.0, 6.4) | (-128.2, 425.6) | (0.0, 6.4) |
| Quintile 1 | (-156.4, 362.0) | (0.2, 3.0) | (-156.4, 364.4) | (0.2, 6.7) | (-156.4, 362.0) | (0.2, 7.2) | (-156.4, 362.0) | (0.0, 6.4) | (-156.4, 364.4) | (0.0, 6.7) |
| Quartile 1 | (-175.5, 335.4) | (0.1, 2.8) | (-170.7, 237.8) | (0.1, 2.1) | (-175.5, 335.4) | (0.1, 8.0) | (-167.6, 246.4) | (0.1, 6.2) | (-170.7, 237.8) | (0.1, 7.8) |
| Tertile 1 | (-180.5, 260.0) | (0.1, 5.1) | (-177.4, 245.6) | (0.1, 2.7) | (-180.5, 257.0) | (0.1, 5.1) | (-177.4, 261.3) | (0.1, 5.5) | (-178.4, 252.4) | (0.1, 5.9) |
| **Propensity Weighting**^¶^ | | | | | | | | | | |
| ATT | (-165.7, 237.3) | (1.1, 16.7) | (-195.2, 221.1) | (4.8, 14.7) | (-164.6, 237.6) | (1.1, 16.8) | (-125.2, 243.4) | (0.6, 24.3) | (-193.0, 222.5) | (2.6, 14.6) |
| ATE | (-139.3, 190.6)  Mean:105.2 | (1.0, 12.4) | (-139.8, 185.5)  Mean: 113.7 | (1.9, 12.7) | (-138.9, 190.4)  Mean: 105.2 | (1.0, 12.5) | (-125.6, 185.4)  Mean: 111.4 | (0.6, 15.9) | (-139.0, 185.5)  Mean: 113.9 | (1.2, 12.9) |
| **^†^**Range across all covariates (not only covariates in the propensity score model); covariates included in each model are described below.  **^‡^**Greedy match: experimentally infected were selected randomly, one by one and matched with the observation in the naturally infected group that had the nearest linear propensity score.  **^§^**Bottom quantiles reported only: Only bottom quantiles for stratification by sextiles, quintiles, quartiles, and tertiles had enough samples in both groups to take standardized differences and variance ratios for all covariates.  ^¶^ATT. Average treatment effect for the treated (where naturally infected cases are considered “treated”); Propensity weighting by ATE (average treatment effect approach, where naturally infected cases are considered “treated”) consistently provided the best balance between groups. For this reason, mean absolute standardized differences are given to provide an additional indicator of balance beyond standardized difference ranges.  **Model 1 covariates:** lower respiratory score, cough score, cough count  **Model 2 covariates:** lower respiratory score, cough score, cough count, upper respiratory score  **Model 3 covariates:** lower respiratory score, cough score, cough count, NP swab Ct value  **Model 4 covariates:** lower respiratory score, cough score, cough count, body temperature  **Model 5 covariates:** lower respiratory score, cough score, cough count, upper respiratory score, nasopharyngeal swab Ct value | | | | | | | | | | |

**Table S5 (part 2/3). Quality of covariate balance after propensity score adjustment**

|  | **Model 6** | | **Model 7** | | **Model 8** | | **Model 9** | | **Model 10** | |
| --- | --- | --- | --- | --- | --- | --- | --- | --- | --- | --- |
| **Approach** | **Standardized differences** | **Variance ratios** | **Standardized differences** | **Variance ratios** | **Standardized differences** | **Variance ratios** | **Standardized differences** | **Variance ratios** | **Standardized differences** | **Variance ratios** |
| **No adjustment** | (-125.6, 288.9) | (0.7, 19.2) | (-125.6, 288.9) | (0.7, 19.2) | (-125.6, 288.9) | (0.7, 19.2) | (-125.6, 288.9) | (0.7, 19.2) | (-125.6, 288.9) | (0.7, 19.2) |
| **1:1 Propensity Matching** | (-278.5, 126.0) | (0.3, 8.4) | (-287.8, 126.0) | (0.3, 8.4) | (-385.5, 132.9) | (0.2, 8.4) | (-557.0, 124.9) | (0.2, 7.4) | (-421.7, 132.9) | (Inf, 8.4) |
| **Propensity subclassification by stratification** | | | | | | | | | |  |
| Sextile 1 | (-128.2, 425.6) | (0.0, 1.2) | (-128.2, 425.6) | (0.0, 1.2) | (-166.9, 319.8) | (0.0, 5.2) | (-129.3, 505.3) | (0.0, 3.4) | (-147.3, 406.5) | (0.0, 5.9) |
| Quintile 1 | (-153.7, 218.9) | (0.1, 3.3) | (-156.4, 364.4) | (0.0, 2.4) | (-151.9, 341.6) | (0.0, 3.7) | (-144.0, 401.8) | (0.0, 10.1) | (-167.0, 435.9) | (0.0, 7.9) |
| Quartile 1 | (-166.0, 237.8) | (0.1, 3.0) | (-166.0, 237.8) | (0.3, 3.1) | (-174.4, 334.2) | (0.0, 4.3) | (-158.7, 364.1) | (0.0, 9.7) | (-174.4, 348.0) | (0.0, 6.1) |
| Tertile 1 | (-177.4, 250.2) | (0.1, 2.5) | (-175.6, 242.1) | (0.1, 3.2) | (-185.3, 335.6) | (0.1, 6.4) | (-174.4, 361.4) | (0.0, 8.7) | (-186.1, 276.2) | (0.1, 6.4) |
| **Propensity Weighting** | | | | | | | | | | |
| ATT | (-163.3, 232.8) | (0.4, 23.5) | (-157.8, 133.8) | (0.6, 56.1) | (-164.8, 233.4) | (0.7, 15.8) | (-107.8, 240.4) | (0.3, 18.5) | Only 1 experimental case used | Only 1 experimental case used |
| ATE | (-130.5, 184.4)  Mean:121.8 | (1.0, 14.9) | (-128.5, 179.0)  Mean: 123.1 | (0.9, 17.2) | (-136.3, 177.6)  Mean: 104.1 | (1.0, 12.3) | (-101.1, 169.3)  Mean: 101.4 | (1.1, 17.1) | (-117.6, 179.4)  Mean: 104.0 | (0.9, 21.2) |
| **Model 6 covariates:** lower respiratory score, cough score, cough count, upper respiratory score, body temperature  **Model 7 covariates:** lower respiratory score, cough score, cough count, upper respiratory score, NP swab Ct value , body temperature  **Model 8 covariates:** cough and cough count  **Model 9 covariates:** cough count and body temperature  **Model 10 covariates:** cough, cough count and body temperature | | | | | | | | | | |

**Table S5 (part 3/3). Quality of covariate balance after propensity score adjustment**

|  | **Model 11** | | **Model 12** | | **Model 13** | | **Model 14** | |
| --- | --- | --- | --- | --- | --- | --- | --- | --- |
| **Approach** | **Standardized differences** | **Variance ratios** | **Standardized differences** | **Variance ratios** | **Standardized differences** | **Variance ratios** | **Standardized differences** | **Variance ratios** |
| **No adjustment** | (-125.6, 288.9) | (0.7, 19.2) | (-125.6, 288.9) | (0.7, 19.2) | (-125.6, 288.9) | (0.7, 19.2) | (-125.6, 288.9) | (0.7, 19.2) |
| **1:1 Propensity Matching** | (-363.5, 132.9) | (0.23, 8.4) | (-574.5, 129.5) | (0.4, 78.3) | (-342.7, 129.5) | (0.2, 9.4) | (-295.9, 110.1) | (0.2, 11.2) |
| **Propensity subclassification by sextiles** | | | | | | | | |
| Sextile 1 | (-158.1, 329.9) | (0.0, 4.9) | (-212.1, 348.9) | (0.2, 5.1) | (-188.3, 203.6) | (0.2, 3.0) | (-200.1, 240.5) | (0.0, 9.1) |
| Quintile 1 | (-171.4, 350.9) | (0.0, 4.3) | (-173.8, 358.2) | (0.1, 10.1) | (-188.8, 204.9) | (0.1, 4.7) | (-161.1, 236.2) | (0.1, 3.4) |
| Quartile 1 | (-175.8, 256.4) | (0.1, 4.7) | (-173.8, 358.2) | (0.1, 10.1) | (-192.1, 208.7) | (0.1, 4.7) | (-174.2, 245.1) | (0.1, 4.4) |
| Tertile 1 | (-152.5, 233.0) | (0.2, 5.1) | (-186.1, 334.3) | (0.1, 8.0) | (-159.2, 251.4) | (0.1, 22.2) | (-139.9, 289.3) | (0.3, 4.9) |
| **Propensity Weighting** | | | | | | | | |
| ATT | (-164.2, 182.8) | (1.2, 59.1) | (-81.7, 263.1) | (0.8, 40.1) | (-84.7, 358.4) | (0.8, 78.9) | (-41.8, 187.6) | (0.3, 515.1) |
| ATE | (-131.1, 176.6)  Mean: 99.4 | (0.9, 24.0) | (-76.3, 178.0)  Mean: 98.8 | (0.5, 35.9) | (-66.9, 169.1)  Mean: 90.6 | (0.4, 48.5) | (-84.2, 166.2)  Mean: 109.1 | (0.1, 30.5) |
| **Model 11 original covariates:** lower respiratory score, age, cough, cough count, upper respiratory score, systemic symptom score, NP Ct value, body temperature (Algorithm did not converge, so chose new model 11 covariates)  **Model 11 new covariates:** cough score, body temperature (Variables were dropped from the original model 11 specification one by one, in order of lowest effect strength to highest; algorithm failed to converge until I arrived at the new model 11 specification)  **Model 12 covariates:** ever fever >37.9^o^C and body temperature  **Model 13 covariates:** ever fever >37.9^o^C, body temperature, and upper respiratory symptom score  **Model 14 covariates:** ever fever >37.9^o^C, body temperature, and total symptom score (Adding cough count to model 14 did not improve the population balance after ATE weighting.) | | | | | | | | |

**Table S6. Covariates with propensity score ATE weighted adjustment**

| **Variables** | **Experimental**  **N=2.66** | **Natural**  **N=67.84** | **Standardized Difference %** | **P value**  **(parametric) ^†^** | **P value**  **(non-parametric) ^‡^** |
| --- | --- | --- | --- | --- | --- |
| **Age** | 25.37 (5.94) | 21.73 (6.49) | -57.0 | 0.119 | 0.066 |
| **Participants with fever >37.9^o^C (%)** | 2.01 (75.4) | 28.70 (42.3) | -66.9 | 0.124 | 0.124 |
| **Temperature (C)** | 37.55 (0.60) | 37.42 (0.65) | -21.2 | 0.582 | 0.536 |
| **Upper respiratory score** | 37.55 (0.60) | 37.42 (0.65) | -21.2 | 0.582 | 0.536 |
| **Lower respiratory score** | 3.90 (0.96) | 6.72 (2.80) | 110.3 | 0.000 | 0.322 |
| **Systemic symptom score** | 0.25 (0.45) | 3.27 (1.40) | 163.5 | 0.000 | 0.013 |
| **Total symptom score** | 1.57 (0.91) | 5.76 (2.17) | 155.4 | 0.000 | 0.031 |
| **Cough score** | 5.72 (1.45) | 15.74 (4.81) | 161.8 | 0.000 | 0.009 |
| **Cough count** | 0.25 (0.45) | 2.28 (0.77) | 169.1 | 0.000 | 0.056 |
| **Nasopharyngeal swab Ct value** | 1.85 (3.97) | 28.13 (27.62) | 109.1 | 0.000 | 0.146 |
| **Propensity score** | 22.32 (1.42) | 22.64 (5.97) | 7.3 | 0.670 | 0.775 |
| **Linear propensity score** | 0.85 (0.25) | 0.90 (0.15) | 26.6 | 0.534 | 0.536 |
| Reporting mean (standard deviation) for each variable except for fever, from N=11 experimental and N=71 natural cases (selected during maximal aerosol shedding day). The mean and max absolute value standardized differences were 90.6 % and 169.1%, respectively. Weighted ATE adjustment used data from 2.66 experimental and 67.84 naturally infected cases. Kolmogorov-Smirnov test for the mean standardized difference was 0.609 (p<0.001). Sex was removed because no females shed into aerosols in the experimental group. **^†^**t-tests; **^‡^**Kolmogorov-Smirnov tests | | | | | |

**Figure S3.** Nasopharyngeal swab Ct values over time. Includes all qRT-PCR detectable nasopharyngeal swab samples during days 1-6 post-inoculation (N=179), and days 1-3 post symptom onset (N=143) for experimental and naturally infected cases, respectively. Boxes show the inner-quartile range (IQR) with a band to indicate the median, and whiskers extending to the highest and lowest data points within 1.5 IQR.

**Figure S4.** Maximum fine and coarse aerosol shedding, limited to samples above LOD (N=11 experimental and N=71 natural), (**A**) histogram of RNA copies shed into fine and coarse aerosols, (**B**) RNA shedding comparison by group and aerosol fraction, (**C**) RNA shedding quantity of samples by quantile for each group and aerosol fraction.

**SI References**

1. Yan J, Grantham M, Pantelic J, et al. Infectious virus in exhaled breath of symptomatic seasonal influenza cases from a college community. Proc Natl Acad Sci U S A 2018;115:1081-1086.

2. Gabriel G, Arck PC. Sex, Immunity and Influenza. J Infect Dis 2014;209:S93-S99.

3. Nguyen-Van-Tam JS, Killingley B, Enstone J, et al. Minimal transmission in an influenza A (H3N2) human challenge-transmission model with exposure events in a controlled environment. medRxiv December 2019:2019.12.13.19014381.

4. Sobel Leonard A, McClain MT, Smith GJD, et al. Deep Sequencing of Influenza A Virus from a Human Challenge Study Reveals a Selective Bottleneck and Only Limited Intrahost Genetic Diversification. J Virol 2016;90:11247-11258.

5. Alford RH, Kasel JA, Gerone PJ, Knight V. Human influenza resulting from aerosol inhalation. Proc Soc Exp Biol Med Soc Exp Biol Med N Y N 1966;122:800-804.

6. Keitel WA, Couch RB, Cate TR, Six HR, Baxter BD. Cold Recombinant Influenza B/Texas/1/84 Vaccine Virus (CRB 87): Attenuation, Immunogenicity, and Efficacy against Homotypic Challenge. J Infect Dis 1990;161:22-26.

7. Austin PC. The Relative Ability of Different Propensity Score Methods to Balance Measured Covariates Between Treated and Untreated Subjects in Observational Studies. Med Decis Making 2009;29:661-677.

8. Austin PC, Grootendorst P, Anderson GM. A comparison of the ability of different propensity score models to balance measured variables between treated and untreated subjects: a Monte Carlo study. Stat Med 2007;26:734-753.

9. Brookhart MA, Schneeweiss S, Rothman KJ, Glynn RJ, Avorn J, Stürmer T. Variable selection for propensity score models. Am J Epidemiol 2006;163:1149-1156.

10. Rubin DB. Using Propensity Scores to Help Design Observational Studies: Application to the Tobacco Litigation. Health Serv Outcomes Res Methodol 2001;2:169-188.

11. Onodera T, Hosono A, Odagiri T, et al. Whole-Virion Influenza Vaccine Recalls an Early Burst of High-Affinity Memory B Cell Response through TLR Signaling. J Immunol 2016;196:4172-4184.

12. Berghöfer B, Frommer T, Haley G, Fink L, Bein G, Hackstein H. TLR7 Ligands Induce Higher IFN-α Production in Females. J Immunol 2006;177:2088-2096.

13. Fischinger S, Boudreau CM, Butler AL, Streeck H, Alter G. Sex differences in vaccine-induced humoral immunity. Semin Immunopathol 2019;41:239-249.
